# Development and implementation of an interdisciplinary one-stop clinic for infant hip dysplasia: A pilot study

**DOI:** 10.1016/j.clinsp.2026.100864

**Published:** 2026-02-12

**Authors:** Patricia Moreno Grangeiro, Giovanna Braga Motta, Natasha Vogel Majewski Rodrigues, Daniel Frank Liggio, Cristiane Aun Bertoldi, Roberta Coimbra, Ina Fialho Zürcher, Yasmin Estefanía González Herrera, Moisés de Freitas Laurentino, Lisa Suzuki, Valdenise Tuma Calil, Nei Botter Montenegro, Giovanni Guido Cerri, Tarcísio Eloy Pessoa de Barros Filho, Werther Brunow de Carvalho, Roberto Guarniero

**Affiliations:** a Instituto de Ortopedia e Traumatologia, Hospital das Clínicas HCFMUSP, Faculdade de Medicina, Universidade de São Paulo, São Paulo, SP, Brazil; b Rutgers New Jersey Medical School, Newark, NJ, USA; c Faculdade de Arquitetura e Urbanismo e de Design, Universidade de São Paulo, São Paulo, SP, Brazil; d Instituto de Radiologia, Hospital das Clínicas HCFMUSP, Faculdade de Medicina, Universidade de São Paulo, São Paulo, SP, Brazil; e Instituto da Criança e do Adolescente, Hospital das Clínicas HCFMUSP, Faculdade de Medicina, Universidade de São Paulo, São Paulo, SP, Brazil

**Keywords:** DDH, One-stop clinic, Graf method, Screening, Flowchart

## Abstract

**Objectives:**

The authors hypothesized that establishing a one-stop clinic for the management of Developmental Dysplasia of the Hip (DDH) at our institution would enhance screening procedures, referral processes, patient compliance, early diagnosis, and timely treatment for this condition. To inform clinical practice, the authors conducted a collaborative development of flowcharts for evidence-based patient care protocols, based on the Graf method and patient journey optimization.

**Methods:**

A weekly DDH clinic was established in a tertiary/quaternary public teaching hospital in Brazil, integrating pediatrics, radiology, and orthopedics. Flowcharts were collaboratively designed during ten interdisciplinary meetings. As a pilot study, the authors included an initial cohort involved in an observational retrospective study during the period from October 2023 to December 2024.

**Results:**

A novel system of grouping Graf types and subtypes was presented, accompanied by a treatment algorithm and phased therapeutic approach, based on the underlying hip pathology and immediate therapeutic requirements to promote hip centralization and acetabular development. A total of 98 infants were examined at the clinic during the specified period, with 22 receiving non-surgical treatment utilizing the Pavlik harness.

**Conclusion:**

The establishment of a one-stop DDH clinic demonstrated a successful interdisciplinary approach, contributing to early diagnosis and effective treatment of DDH. The collaborative development of flowcharts facilitated the standardization of patient management protocols. Continued refinement of screening procedures and data collection methods is necessary to optimize care and inform future research on DDH management.

## Highlights

• Describes the co-design and pilot implementation of a one-stop clinic for DDH.
• Introduces a novel grouping of Graf subtypes to guide a structured three-phase treatment.
• Presents collaboratively designed algorithms for clinic workflow and evidence-based treatment.
• In the pilot phase, 22 of 98 infants were treated with a Pavlik harness, showing feasibility.
• Aims to standardize care and serve as a model for implementation in similar healthcare settings.

## Introduction

Developmental Dysplasia of the Hip (DDH) is a neonatal condition characterized by a hip that may be dislocated (either reducible or irreducible), unstable, or stable but with insufficient acetabular coverage.^1^ Dislocations and instability can be detected through a clinical examination, whereas a stable hip with insufficient coverage typically requires additional imaging, most commonly via ultrasonography.^2^, ^3^, ^4^ The existence of cases that can only be identified through imaging has led to debates about the most effective screening methods. Screening strategies include universal clinical screening followed by either universal or selective ultrasound screening.^5^ In that way, the ultrasound technique represents a crucial quality factor that should not be ignored. The Graf method is widely regarded as the most reproducible and practical ultrasound examination technique; when the prescribed procedures are meticulously adhered to, it facilitates accurate diagnosis and informs treatment decisions.^6^^,^^7^

Screening aims to stratify a population by identifying those at an increased risk of disease, helping detect disease in its early stages, and allowing timely intervention to prevent disease progression.^8^ The risk factors of breech presentation and family history, among others under investigation, have been indicated as important in screening for DDH.^9^ Early diagnosis of DDH enables effective non-surgical management using braces or orthoses, which has shown high efficacy and favorable long-term outcomes.^10^ In contrast, delayed diagnosis may make nonsurgical interventions impractical, leading to the need for more complex and high-risk surgical treatments.^11^

The implementation of an efficient and effective public health program for the management of DDH in infants requires careful consideration of multiple interconnected phases. These phases include screening, referral, diagnosis, and treatment, all of which are crucial for ensuring that patients receive appropriate care. Each phase should be planned and executed with attention to its interdependence. As of now, there are no comprehensive descriptions of the entire spectrum of non-surgical DDH care, from initial screening to non-operative treatment, within the Brazilian medical literature^12^. Variability in clinical practice and the limited availability of evidence-based treatment guidelines impede the development of a consistent practice model regarding non-operative treatment of DDH.

The authors hypothesized that establishing a one-stop clinic at our institution, a tertiary/quaternary public teaching hospital in Brazil, would improve screening, referrals, patient adherence, early diagnosis, and timely treatment for DDH. Moreover, in this model, patients can receive clinical, sonographic, and radiographic evaluations in the same location at the same appointment, which could enhance care standards while reducing costs for both the healthcare system and patients.^5^

One of the first steps in bringing together the interdisciplinary team involved collaboration. In addition to the clinical responsibilities, the team came together to create patient care flowcharts based on evidence from the literature and established healthcare practices. The primary objectives of this study were to: 1) Collaboratively design flowcharts for clinical care and patients’ journey and 2) Gather data from a retrospective chart review during the implementation phase as a pilot study design to support the development of a prospective study.

## Methods

### Study design

This study documents the establishment of a dedicated weekly DDH clinic for screening, referral, diagnosis, and management of this condition in neonates and infants up to 6 months of age in the studied institution, a tertiary/quaternary public teaching hospital in Brazil. This was a collaborative effort among the departments of pediatrics, radiology, and orthopedics of the University of São Paulo Faculty of Medicine, and the University of São Paulo Faculty of Architecture and Urbanism and Design (specialized in healthcare information design). During the study period, clinic flow and patient care flowcharts and a database for continuous implementation were developed. The initial cohort served as data for a pilot study, collected as an observational retrospective chart review during the period from October 2023 to December 2024. The study received approval from the institution’s Ethics Committee (CAAE 37,126,920.9.0000.0068).

### Screening and referral of patients

Patients were referred by neonatologists from the maternity ward who were responsible for the care of newborns in the hospital. For this phase, they were instructed to refer infants who, on clinical examination, demonstrated positive results on the Ortolani or Barlow maneuvers, or those with positive risk factors, such as a breech presentation or a family history of DDH. Only patients born without a concurrent neuromuscular disorder or other known conditions were referred to this specific clinic. Any twins of the eligible infants were also referred for further screening.

Patient referrals were conducted through the hospital's reference system and directly via electronic messaging. As the clinic was in the implementation phase, neonatologists documented risk factors and any positive signs on the clinical exam without a standard written form. The maternity unit at this institution specializes in high-risk pregnancies, resulting in a high incidence of twin births.

### Clinic setting and context

The clinical environment was designed as a comprehensive care facility where patients could receive orthopedic care, ultrasound examinations, and radiographic imaging during a single appointment in the same location. Patients were referred from the maternity department to this clinic located within the orthopedic building of the hospital complex.

The clinic was committed to both patient care and medical education; it included an orthopedic team consisting of the lead pediatric orthopedic surgeon, fellows, and residents, who focused solely on assessing patients with suspected or confirmed DDH. A radiology team, made up of an attending physician, fellows, and residents, was headed by a pediatric radiologist with more than two decades of experience in teaching and training in the ultrasonographic Graf method.

**Fig. 1:**
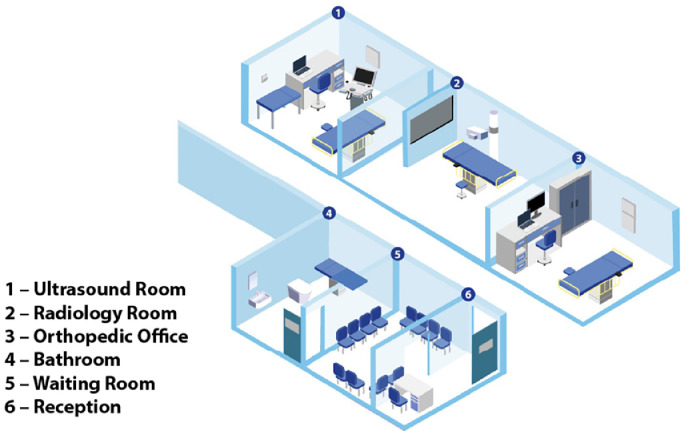
Clinic setting.

The clinic was structured to facilitate optimal access to all requisite diagnostic and treatment resources. The rooms designated for orthopedic, ultrasound and radiographic examinations were situated within the same corridor; patients could easily navigate between the different rooms, and healthcare professionals could quickly share and discuss cases (Fig. 1). Additionally, patients and caregivers were provided with a dedicated waiting area, and provisions were made to accommodate nursing and diaper changes.

Initially, patients underwent a comprehensive orthopedic and hip examination, then were directed to diagnostic imaging with radiologists, should it be indicated at that visit or scheduled for imaging at a more appropriate age. Within the context of this clinic, the orthopedic surgeon held responsibility for the clinical management and oversight of the patients.

### Diagnosis

#### Ultrasound physical setting and positioning

Ultrasound examinations were conducted following the Graf method. An ultrasound device (LOGIQ-9, GE Healthcare, Wisconsin, USA) and a 9 Mhz linear transducer were utilized. The infant stayed in a physiological state of comfort in the lateral decubitus position. The hip was slightly flexed, and with a spontaneous position of the lower limb, was gently brought together. (Note: at the time of this study, the clinic had not yet obtained a positioning Graf cradle or its transducer guide, which are recommended for hip examinations using the Graf method, as they assist in the positioning of the patient and the training of examiners. Nevertheless, the final image was deemed accurate once the checklists were completed and the measurement technique applied to the image was verified as appropriate. For each hip examined, the examiner was required to capture and document at least two images taken at different intervals.

### Graf method and measurement techniques

The Graf method consists of obtaining a coronal image of the hip, in a standard plane, which is then subjected to an anatomical evaluation, a measurement of angles, and, in hips classified as IIc, the image is repeated as pressure is applied (a modified Barlow maneuver).^6^ The standard plane ensures adequate positioning through the center of the acetabulum, with the pivotal point being the lower limb of the os ilium. A plane requires three coordinates (landmarks), which specifically are: the lower limb of the os ilium, the midsection of the bony acetabular roof, and the acetabular labrum

**Fig. 2:**
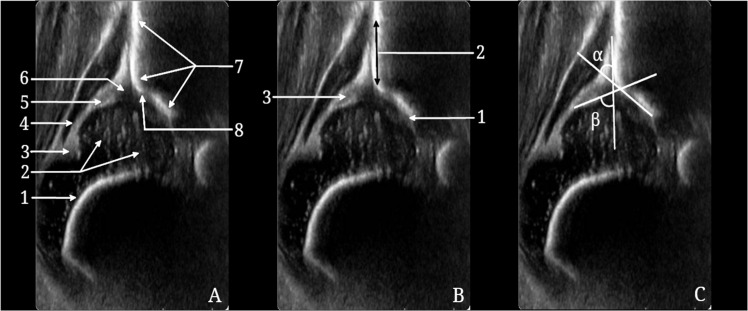
An ultrasound image of the hip showing Checklist 1, Checklist 2, and the measurement of angles in the Graf method. (A) Checklist 1 for anatomy: 1 - Chondro-osseous junction, 2 - Femoral head, 3 - Synovial fold, 4 - Joint capsule, 5 - Acetabular labrum, 6 - Hyaline cartilage roof, 7 - Bony acetabular roof, 8 - Bony rim: turning point from concavity to convexity. (B) Checklist 2 for image plane: 1 - Lower limb of the ilium, 2 - Standard sectional plane, 3 - Acetabular labrum. (C) Alpha and beta angles.

To ensure a comprehensive hip view, specific anatomical landmarks had to be visible. Images were evaluated using Checklist 1 for anatomical perspective and Checklist 2 for location within the acetabular cavity[6] (Fig. 2). Images not meeting these criteria were not measured, except for dislocated hips, where standard plane acquisition was not possible. Sonographic images of either side were optimally interpreted when presented as a right hip in an upright position (standardized method of representation by Graf), akin to radiographic imaging.

After obtaining an adequate image, the alpha and beta angles were measured. The alpha angle (bony roof) is formed by the baseline (a line tangent to the straight echo of the os ilium) and the bony roof line (a line drawn from the lower limb of the ilium tangential to the bony roof). The beta angle (cartilaginous roof) is formed between the baseline and the cartilaginous roof line, which is drawn from the turning point (where the socket's concavity turns to the iliac bone's convexity) to the center of the labrum. The 3 lines are rarely intersected at the same point. The bony rim area, the lateral region of the bony roof, is classified by its morphology as angled, blunt, rounded, or flat.

### Graf classification system

**Fig. 3:**
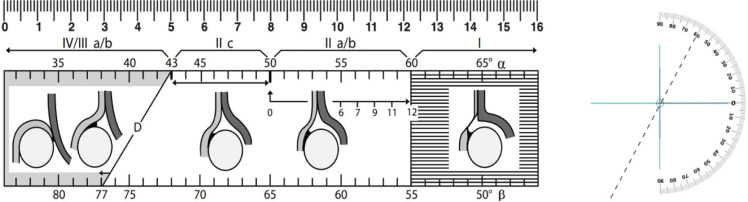
Ruler and sonometer according to Graf.

The Graf classification system initially divides hips into four main types (I–IV) according to the alpha and beta angles. The sonometer (Fig. 3) is a graphical tool that relates the alpha angle to the Graf types. Between 6 and 12 weeks of age, it also incorporates the child’s age in weeks, which is essential for distinguishing physiological (IIa+) from pathological (IIa-) hips. The sonometer is segmented into three intervals based on the alpha angle: the right interval (> 60°, Type I), the central interval (43–59°, Type II), and the left interval (≤ 42°, corresponding to decentered hips, Types III and IV). Type I corresponds to normal hips, whereas Type II indicates dysplasia. Alpha angle thresholds are expected to increase progressively (52° at 4-weeks, 55° at 6-weeks, 57° at 8 = weeks, 58° at 10-weeks). Failure to reach these values indicates Type IIa-. At 12-weeks, an alpha angle below 60° leads to reclassification as Type IIb, which is pathological. The distinction between Types III and IV depends on the position of the hyaline cartilage roof ‒ upward in Type III and downward in Type IV ‒ and is determined by the configuration of the cartilage and the perichondrium outline rather than acetabular roof deformity alone. In summary, diagnosis integrates angular measurements, hip morphology, and the child’s age.

### Novel Graf-based grouping and management protocol

In our protocol, the 11 Graf sonotypes were grouped into five categories, each with a defined management pathway. The structure flows naturally: first by categories, then distinguishing hips not requiring treatment, and finally detailing the three-phase protocol for hips requiring treatment.

• Normal hips (Graf Ia and Ib) – Fully developed hips that required no treatment. Patients were scheduled for radiographic follow-up at 6-months, 1-year, and 2-years to confirm normal acetabular development.
• Immature hips (Graf IIa+) – Physiologically immature hips reassessed at 3 months with clinical examination and ultrasound. If maturation was confirmed, follow-up radiographs were performed as in the normal group.
• Dysplastic hips – managed according to a three-phase protocol:
  Centered stable hips (Graf IIa-, IIb, IIc stable)
Maturation Phase: Pavlik harness is used for 23 h/day (one hour off for hygiene/exercises), with continued imaging until normalization (Graf I).
  Centered unstable hips (Graf IIc unstable)
Retention Phase: Pavlik harness is maintained 24 h/day to stabilize the hip and promote acetabular growth. Regular ultrasound and radiographs confirmed stability.
  Decentered hips (Graf D, IIIa, IIIb, IV)
Reduction Phase: The Pavlik harness is prescribed 24 h/day to achieve and maintain a reduction. Ultrasound-guided adjustments and confirmed reduction.

### Co-design of flowcharts

Ten meetings were held with 13 team members to plan and define the work. In the first meeting, two design researchers proposed a 75-minute co-creative workshop to initiate collaborative design of the clinical decision chart, the treatment flowchart, and the hospital care pathway. Based on this workshop, they produced the first iteration of the flowchart. Data compilation was organized on the MIRO platform (http://www.miro.com), which supported iterative design with multiple rounds of prototyping, testing, and validation. The visual flowcharts allowed members to confirm shared understanding, detect ambiguities or errors, and reflect on implementation and monitoring criteria. A chromatic code was created to distinguish stages of the care pathway in infographics and visual representations of the Graf classification system, grouping, and treatment. Real-time editing enabled adjustments in element order, text, process details, and patient inclusion criteria.

### Design of a prospective database

Preliminary findings, including the variables collected and analyzed in this study, were used to build the REDCap[14] database that will support a prospective study aimed at improving the understanding of screening, diagnosis, and treatment of DDH, as well as evaluating the effectiveness of this one-stop clinic. The REDCap platform will ensure future standardized and harmonized data collection.

#### Pilot study data collection

Data were extracted from the medical records of patients who were referred from the maternity ward, admitted, and treated in the clinic for over 15-months, from October 2023 to December 2024. All data were collected and uploaded to Google Sheets (Google LLC, Mountain View, California, USA), and statistical analysis was conducted using the same program. Data collected from the medical record included: number of patients born in the maternity ward during the period, number of patients and reasons listed for referral from the maternity ward, date of referral, date of first appointment, patient demographic data, the results of the orthopedic clinical exam, dates of all exams, imaging results, patients treated, and treatment results. The authors utilized the Strengthening the Reporting of Observational Studies in Epidemiology (STROBE) guidelines for reporting inclusion, exclusion, and those analyzed for the outcome of treatment.^15^

## Results

### Graf classification – based clinical decision chart

**Fig. 4:**
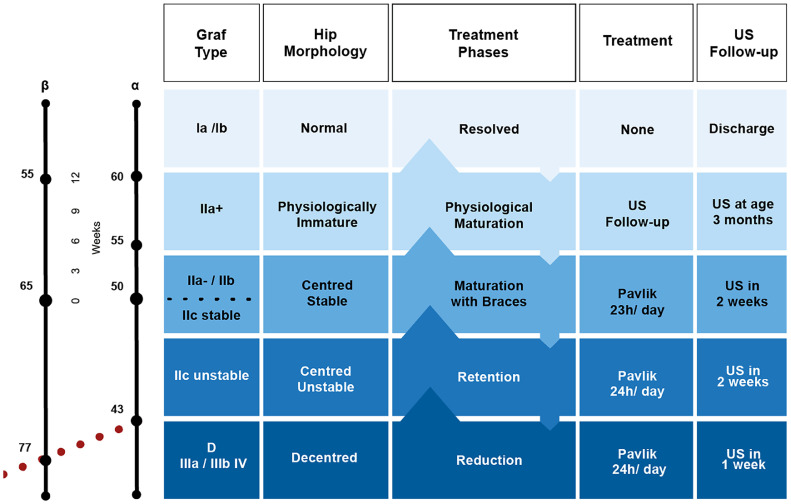
Clinical decision chart for non-operative treatment protocol following the grouping of Graf’s types and subtypes. Graf´s types Ia and Ib are considered normal and do not require treatment. Physiologically immature: Graf type IIa+ are reevaluated at 3-months of age (clinical and ultrasound). Centered stable (Graf type IIa- and IIb, IIc stable), centered unstable (Graf type IIc unstable), decentered (Graf type D/IIIa/IIIb/IV) need treatment. (US, Ultrasound). Dysplastic hips are managed through a structured three-phase protocol reduction, retention, and maturation).

The clinical decision chart illustrating the novel Graf-based grouping and management protocol (Fig. 4) was developed through a co-design process with clinicians and designers, representing one of the first tangible results of this study. It translates evidence and best practices into a standardized pathway for hip surveillance and management. The structure flows naturally: hips are first organized into categories; normal and immature hips are outlined as not requiring treatment, while dysplastic hips are managed through a three-phase protocol (reduction, retention, and maturation). This approach ensures both consistency and individualized care, ranging from observation to structured non-operative treatment.

### Flowchart of management protocol

**Fig. 5:**
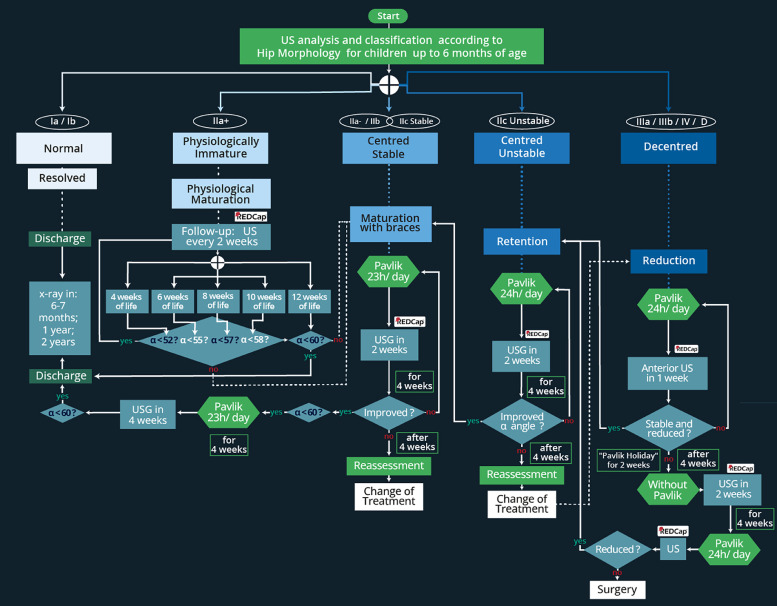
Graf-based treatment flowchart (≤6-months) (US, Ultrasound). Footnote: Normal hips (Ia/Ib) were followed with scheduled radiographic surveillance only. Physiologically immature hips (IIa+) were monitored with ultrasound every 2-weeks; if the alpha angle exceeded 60°, radiographic follow-up was scheduled at 6-months, 1-year, and 2-years. When age-specific thresholds were not met (e.g., α 55° at 8-weeks), hips were reclassified as Iia- and treated as dysplastic. Dysplastic hips (IIa-, IIb, IIc stable, IIc unstable, D, III, IV) were managed through three phases: reduction (decentered hips, Pavlik 24h/day, weekly US), retention (centered unstable hips, Pavlik 24h/day, US every 2-weeks), and maturation (centered stable hips, Pavlik 23h/day, imaging until normalization). If expected progress was not achieved, the protocol mandated reassessment, treatment adjustment, or escalation (e.g., Pavlik holiday, closed reduction under anesthesia).

The flowchart in Fig. 5 expands upon the decision chart by presenting a step-by-step clinical pathway for the surveillance and management of hips up to six months of age, enabling dynamic decision-making at each stage of treatment. This detailed algorithm integrates the timing of reassessments, criteria for progression between phases, and indicators for modifying or discontinuing treatment, thereby providing operational guidance for clinicians. It allows tracking of patient progression through the reduction, retention, and maturation phases, while reinforcing the importance of continuous monitoring and timely reassessment.

For the first group (Graf Ia/Ib hips), the management pathway remains as outlined in the clinical decision chart, consisting solely of scheduled radiographic surveillance. The second group included hips classified as physiologically immature. For these patients, age-specific alpha angle thresholds were applied, recognizing that an alpha angle of 55° is acceptable at 4-weeks but considered dysplastic at 8-weeks, necessitating intervention. Between 6- and 12-weeks, type II hips are further classified as IIa- or IIa+, depending on the alpha angle achieved. Hips that remained IIa+ were followed with ultrasound every two weeks until the alpha angle exceeded 60°, at which point they were scheduled for radiographic evaluations at 6-months, 1-year, and 2-years. Hips classified as IIa-, as well as IIb, were identified as dysplastic and were treated with the Pavlik harness for 23-hours per day and monitored with ultrasound every 2-weeks until the alpha angle exceeded 60°

The final three groups were considered abnormal and required intervention. Decentered hips (Graf types D, IIIa, IIIb, and IV) began the reduction phase with the application of a Pavlik harness worn 24-hours a day, accompanied by weekly anterior ultrasounds to confirm progress toward congruent reduction. Once centered, these hips, along with centered but unstable hips (Graf IIc unstable), progressed to the retention phase, during which the harness continued to be worn 24-hours a day with ultrasound reassessments every 2-weeks to monitor stability. When stability was achieved, these hips, together with those initially classified as centered stable (Graf IIa-, IIb, and IIc stable), entered the maturation phase, during which the Pavlik harness was worn for 23-hours per day, and periodic imaging was performed to ensure proper acetabular development until full normalization was confirmed.

For hips that did not achieve the expected progress during any phase, the protocol mandated timely reassessment and adaptation of treatment. In the reduction phase, if centering was not achieved after three weeks, a Pavlik holiday ‒ a temporary discontinuation of harness use for two weeks ‒ was implemented before resuming treatment. If reduction still failed, escalation to closed reduction under anesthesia with subsequent immobilization was considered. Likewise, in the retention and maturation phases, if improvement in the alpha angle or stability was not demonstrated during scheduled reassessments (typically after four weeks), the treatment plan was revised, with reinforcement of guidance and counseling for parents to ensure adherence and optimize outcomes.

### Flowchart for the continuation of the clinic incorporating prospective data collection by REDCap

**Fig. 6:**
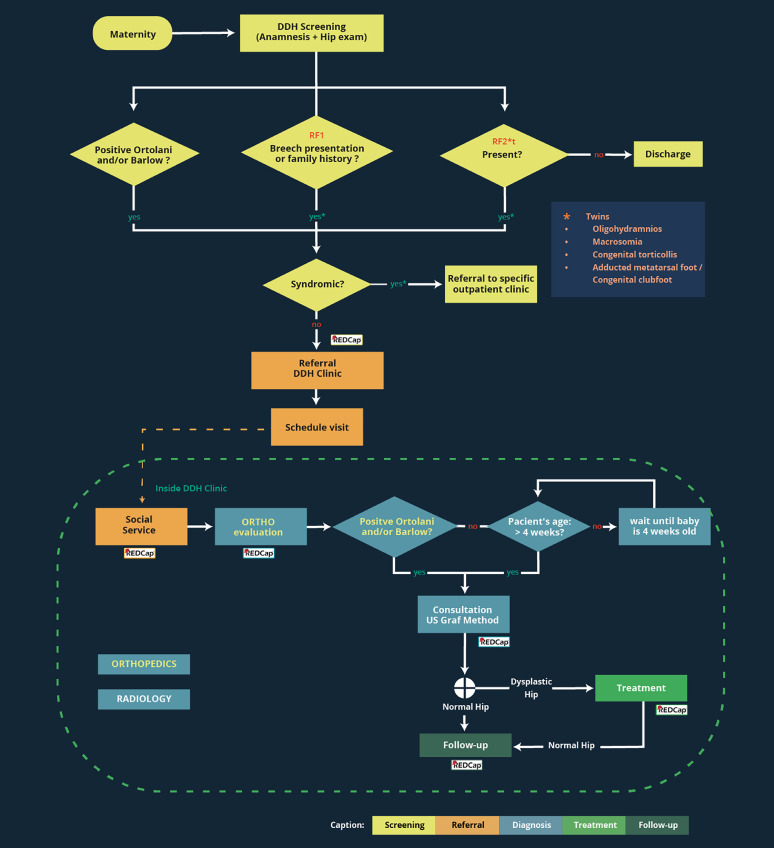
Patient pathway from maternity to the DDH clinic with integrated REDCap data collection. DDH, Developmental Dysplasia of the Hip; RF, Risk Factors.

This flowchart (Fig. 6) illustrates the optimal patient trajectory across hospital units and the standardized procedures within each unit, with color-coding applied to enhance clarity and usability. In the next phase, to support the prospective study, data collection at each step of this pathway will be integrated into the REDCap platform, ensuring structured and secure documentation of clinical and imaging data. Moreover, the protocol will be expanded to incorporate additional risk factors for neonatologists to consider when referring infants, including multiple gestation (twins), oligohydramnios, macrosomia, clubfoot, and metatarsus varus. These additions will contribute to a more comprehensive and inclusive referral process.

### Data for the pilot study

**Fig. 7:**
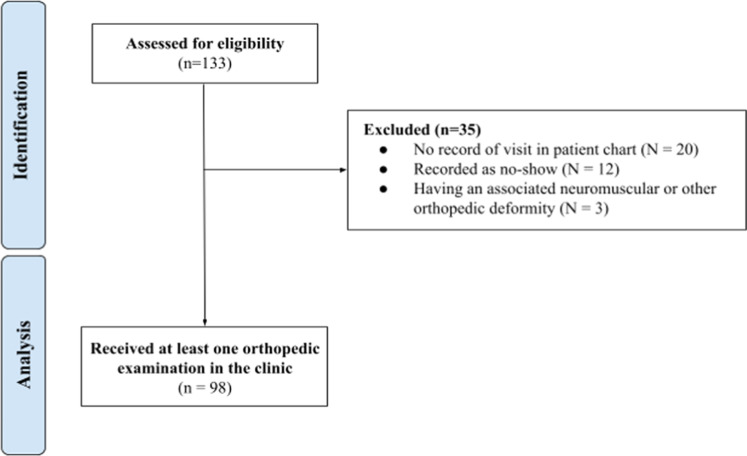
A STROBE diagram showing the process of a patient record retrieval and reasons for exclusion.

A diagram according to the Strengthening the Reporting of Observational Studies in Epidemiology (STROBE) was included and presented in Fig. 7.

According to records from the maternity ward, 1741 children were born during the study period, and 133 were referred to the DDH clinic. Of these, 98 patients who were seen and examined during the study period were suitable for analysis. Among these 98 infants, 22.4 % (22/98 infants, 31 abnormal hips) were diagnosed with DDH and subsequently initiated the treatment protocol with the Pavlik harness, thus being included in the study cohort. Among those who commenced treatment, 54.5 % (12/22 infants, 15 hips) completed bracing treatment, while 0 % [0 %, 24.3 %] (0/12 infants, 0 hips) experienced treatment failure due to one or more irreducible hips. The characteristics of the final study cohort are presented in Table 1.

**Table 1:** Demographic and referral data of patients evaluated at the hip dysplasia clinic.

		n	%
**Sex**	Female	58	59.2
	Male	40	40.8
**Referral Motive**	Ortolani Positive	11	11.2
	Hip Click	6	6.1
	Breech Presentation	32^a^	32.7
	Family History	0	0
	Twin Birth	14	14.3
	Not specified	35	35.7
**Concurrent Orthopedic Conditions**	Congenital torticollis	2	2.0
	Clubfoot	2	2.0
	Preaxial polydactyly	2	2.0
	Metatarsus adducts	1	1.0
	Congenital dislocation of the knee	1	1.0

a One set of twins (two patients) was referred for both breech presentation and twin birth. These two patients are recorded in this table as being referred for breech presentation.

The Graf classification and treatment group of examined patients is shown in Table 2. Two patients seen in the clinic were unable to be examined by the Graf method due to being older and having femoral ossification incompatible with the Graf method. They subsequently underwent examination using plain radiographs instead.

**Table 2:** Graf´s type and treatment group results from the first ultrasound exam of patients seen in the clinic.

Graf Type	n	% [95 % CI]	Treatment Group by Hip Pathology	n	% [95 % CI]
Graf Ia	53	27.60 [21.77, 34.32]	Normal	125	65.10 [58.11, 71.55]
Graf Ib	72	37.50 [30.88, 44.50]			
Graf IIa+	36	18.75 [13.43, 25.20]	Immature	36	18.75 [13.43, 25.20]
Graf IIa-	6	3.13 [1.28, 6.59]	Centered Stable	13	6.77 [3.89 11.08]
Graf IIb	5	2.60 [0.96, 5.96]			
Graf IIc Stable	2	1.04 [0.26, 3.70]			
Graf IIc Unstable	13	6.77 [3.89, 11.08]	Centered Unstable	13	6.77 [3.89, 11.08]
Graf D	4	2.08 [0.68, 5.25]	Decentered	5	2.60 [0.96, 5.96]
Graf IIIa	1	0.52 [0.09, 2.87]			
Graf IIIb	0	0 [0.00, 1.96]			
Graf IV	0	0 [0.00, 1.96]			
Total	192	100	Total	192	100

*The one case of residual dysplasia after conservative treatment occurred six months after completion of treatment with a Pavlik harness and on the third post-treatment screening X-Ray. Treatment continued with a Milgram brace.

## Discussion

This article reports on a pilot experience with the inauguration of a weekly dedicated one-stop DDH clinic in a tertiary university hospital, characterized by an integrated multidisciplinary approach. Beyond the immediate clinical outcomes, the co-designed charts, flowcharts, and the creation of a REDCap database represented key outputs of this phase, providing standardized tools to support decision-making, guide surveillance and treatment, and enable structured data collection. Retrospective data collected during implementation informed the selection of clinical, epidemiological, and radiographic variables for the REDCap database, ensuring its relevance for both patient care and research. These developments are expected to strengthen future research initiatives and facilitate the transition toward larger-scale, potentially multicenter projects.

Screening for DDH presents unique challenges because the condition is insidious, dynamic, and progressive.^16^ The institution adopted a strategy of universal clinical screening combined with selective ultrasound for infants with positive clinical findings and/or risk factors. This approach was preferred over universal ultrasound screening, given the inconclusive evidence supporting the latter and the reduced resource demands of the former.^7^, ^8^, ^9^, ^10^, ^11^, ^12^, ^13^, ^14^, ^15^, ^16^, ^17^, ^18^, ^19^, ^20^ Importantly, such a system requires a robust and reliable referral network. In England, the Newborn and Infant Physical Examination (NIPE) program ensures early recognition and treatment of conditions affecting the eyes, ears, hips, and testes (in males).^21^ In Brazil, the official national protocol mandates neonatal physical screening for the eyes, ears, and heart; hip screening, however, is carried out only according to professional recommendations.

This one-stop clinic was designed to maximize the accurate diagnosis and effective treatment of DDH while minimizing unnecessary visits and examinations, thereby reducing the economic impact on families and the public health system. For this reason, only the two strongest risk factors ‒ positive family history and breech presentation at birth ‒ were initially considered sufficient to warrant a full orthopedic evaluation and ultrasound examination.^18^ In practice, however, 20.4 % of referrals were made for patients with risk factors outside this protocol, and 35.7 % of referrals had no documented reason. Several perinatal factors, such as foot deformities, twin birth, torticollis, and oligohydramnios, are weakly or inconsistently associated with DDH, but the absence of systematic data collection in this clinic limited the ability to analyze their true impact.^5^^,^^22^ Without a standardized referral protocol applied consistently, unnecessary testing may occur, and essential information, such as family history of hip dysplasia, may be overlooked.

Before this initiative, at our institution, infants suspected of having DDH were referred within the Brazilian Unified Health System (Sistema Único de Saúde ‒ SUS). Although effective as a national healthcare system, its decentralized structure often results in fragmented care.^23^ A complex condition such as DDH, requiring the participation of multiple specialists in different facilities and appointments, carries a significant risk of delayed or neglected diagnosis. Indeed, between 2012 and 2016, 17 patients over the age of 2-years at this institution presented with late-diagnosed DDH and required surgical treatment, including open reduction, pelvic osteotomy, and spica immobilization.^24^ The introduction of the one-stop clinic addressed this gap by enabling comprehensive clinical and imaging assessment, as well as treatment planning, within a single appointment. This model has been widely documented in the United Kingdom, where patients undergo both clinical and ultrasonographic or radiographic assessments during the same consultation^25^^,^^26^ ‒ and at the Toronto Hospital for Sick Children.^27^ Its main advantages include reducing the burden of multiple hospital visits on families and minimizing delays in obtaining imaging, making treatment decisions, and initiating management.

Of the numerous methods for ultrasound examination in DDH, the Graf method is recognized as the global standard.^28^ For this clinic, it was adopted for its reliability and accuracy.^3^ Nevertheless, the Graf method is highly technical and strongly dependent on operator training. Ossendorf et al. (2022) reviewed 10 of the 43 studies cited in a systematic review of ultrasound methods for DDH that included actual exam images, and found that only 5 displayed images consistent with Graf’s Checklist 1 and Checklist 2.^29^^,^^30^ This underscores the authors’ experience that the Graf method should be performed and interpreted only by professionals with specialized training. Within the one-stop clinic, all infants are examined by radiologists trained in the Graf method alongside orthopedists with expertise in this imaging technique. The model also facilitates direct communication between radiologists and orthopedists, fostering continuous learning, teaching opportunities, and rapid resolution of any uncertainties regarding images. Centralizing this expertise within the clinic further creates an environment that can support the training of visiting physicians through immersion in the comprehensive care of patients with suspected or confirmed DDH.

The classification system developed by Graf for the interpretation of the infant hip ultrasound exam is highly detailed to categorize all potentially identifiable hip pathologies.^30^ In particular, the subtypes of Graf type II hips have been the subject of discussion and research regarding appropriate treatment protocols.^31^ Many attempts have been made to group the types and subtypes of the Graf classification system to be used to direct treatment.^32^^,^^33^ A grouping of Graf types and subtypes was developed using the experience gained in the development and execution of this one-stop clinic based on expectations for hip development or progression as determined by underlying hip pathology. The flowchart presented in this paper directing treatment is of central importance to the future operation of the one-stop clinic and standardization of treatment.

The goal of the developed treatment protocol is to maximize the likelihood of successful non-surgical management with a Pavlik harness. A scarcity of peer-reviewed, evidence-based treatment guidelines has perpetuated significant practice variation, hindering the standardization of teaching methodologies and limiting the ability to compare outcomes of non-surgical DDH treatment.^34^ Existing guidelines, largely based on expert consensus, generally emphasize early intervention (between 6-weeks and 6-months of age) and continuation until sonographic normalization, with or without gradual discontinuation of the splint.^28^^,^^35^ Overall, long-term outcomes associated with Pavlik harness treatment for DDH are considered favorable.^10^ The treatment guidelines proposed and refined through this clinic are expected not only to guide clinical care but also to inform future research into optimizing treatment strategies, supporting an ongoing process of continuous refinement.

The clinical examination for hip dysplasia remains an area of ongoing study. A recent review by Singh et al. reported that only the Barlow and Ortolani maneuvers demonstrate positive likelihood ratios high enough to be useful in screening for DDH.^36^ This contrasts with other commonly considered findings ‒ such as hip clicks, limited abduction, and asymmetrical thigh folds ‒ which lack clinical utility in screening.^26^^,^^36^ Key historical risk factors consistently identified in the literature include a positive family history of DDH and breech presentation at birth.^9^ As neonatologists are the first physicians to examine newborns, they must be proficient in both the physical exam and recognition of risk factors. At this institution, regular training sessions for neonatologists on updated risk factors and examination techniques are planned. Furthermore, integration of the one-stop clinic with the maternity ward provides neonatologists with the opportunity to observe the entire DDH care pathway and to receive continuous, hands-on training in screening practices.

Among the 1741 children born during the study period, 22 were diagnosed and treated for DDH, corresponding to a prevalence of 1.26 %±0.27 %. The only prior Brazilian study using ultrasound for DDH diagnosis reported a prevalence of 5.46 % in a maternity hospital.^12^^,^^37^ Given that the referring maternity ward at our institution is high-risk, with a large number of breech presentations, and that not all newborns were screened, the true prevalence is likely higher. Treatment outcomes during this pilot phase were excellent. Of the 12 patients who completed treatment with a Pavlik harness, only one case of residual dysplasia was detected on the third follow-up radiograph after harness discontinuation. This was not considered a treatment failure but rather a screening success, as the patient was subsequently managed with a Milgram brace and avoided surgery. Moving forward, treatment in this clinic will be standardized by Graf type according to the flowchart presented in this article, facilitating evaluation of factors associated with treatment success or failure.

### Limitations

The review of the first fifteen months of operation of this clinic highlighted several limitations. The study design was based on a retrospective chart review, resulting in a convenience sample of 98 patients seen and 22 treated, with only short-term follow-up available. This design limited internal validity and reproducibility, but nonetheless provided valuable insights for planning prospective studies and estimating sample size requirements.

A major shortcoming of this pilot phase was the lack of standardized referral data. The direct, contact-based process offered no uniform documentation of referral reasons, prevented comprehensive analysis of referral patterns, and occasionally resulted in data loss when referral emails were misplaced. In addition, caregiver engagement and structured family guidance were not systematically addressed during this initial phase. These limitations, though typical of retrospective studies, underscore the need for standardized and prospective data collection in future research.^38^

### Perspectives for future studies

Despite these limitations, the information obtained during the pilot phase has been instrumental in developing a standardized REDCap database for referrals, diagnoses, and treatment of patients with suspected or confirmed DDH. This will ensure that referral reasons are consistently documented, patient identities are traceable, and longitudinal treatment data can be collected and analyzed.

Another key objective of the clinic is training. Its centralized, high-volume caseload creates an ideal environment for pediatricians, orthopedists, and radiologists to refine their skills in screening, diagnosis, and non-operative treatment of DDH. Moreover, the one-stop clinic model may serve as a reference for scaling to other institutions, offering a practical framework for implementation.

Future research should prioritize prospective data collection, standardized referral protocols, and long-term outcome assessment. Continuous, iterative evaluation of both clinical outcomes and process indicators will be essential to refine the protocol, improve patient engagement, and optimize the effectiveness of the program. Building on the foundations of existing scientific knowledge and clinical practice, the one-stop clinic provides a platform for ongoing innovation in DDH management. The flowcharts and treatment algorithms developed in this pilot phase will serve as key components of an implementation package for future clinics, facilitating adoption and scalability of the model. Ultimately, well-designed multicenter prospective studies will be necessary to generate robust evidence capable of informing public health policies and guiding nationwide strategies for DDH screening and treatment.

## Conclusion

This study aimed to evaluate the first year of operation of a multi-professional one-stop DDH clinic, present screening and treatment algorithms developed from the experience of the clinic’s operation, and present a future direction for the clinic. A novel grouping of Graf types and subtypes was developed along with a treatment algorithm and a phased treatment approach based on the underlying hip pathology and immediate therapeutic need to promote hip centralization and acetabular development. During the first year, 98 patients were seen in the clinic, and 22 received Pavlik harness treatment. A need for greater standardization in practice, training, and data collection methods has been recognized and scheduled for future implementation.

## Data availability

Indication that the data must be requested from the corresponding author.

## Informed consent

As this was a retrospective review of existing medical records and did not involve direct patient contact or the collection of identifiable personal data, the requirement for informed consent was waived by the Ethics Committee.

## Declaration of competing interest

The authors declare no conflicts of interest.
